# Author Correction: Transcriptional characterization of iPSC-derived microglia as a model for therapeutic development in neurodegeneration

**DOI:** 10.1038/s41598-024-60234-z

**Published:** 2024-04-23

**Authors:** Gokul Ramaswami, Yeliz Yuva-Aydemir, Brynn Akerberg, Bryan Matthews, Jenna Williams, Gabriel Golczer, Jiaqi Huang, Ali Al Abdullatif, Dann Huh, Linda C. Burkly, Sandra J. Engle, Iris Grossman, Alfica Sehgal, Alla A. Sigova, Robert T. Fremeau, Yuting Liu, David Bumcrot

**Affiliations:** 1 CAMP4 Therapeutics Corporation, Cambridge, MA USA; 2 Biogen Inc., Cambridge, MA USA; 3 Eleven Therapeutics, Cambridge, MA USA

Correction to: *Scientific Reports* 10.1038/s41598-024-52311-0, published online 25 January 2024

Iris Grossman was omitted from the author list in the original version of this Article.

The Author Contributions section now reads:

“G.R. performed computational analyses and wrote the manuscript draft. Y.Y., B.A., B.M., J.W., and A.A.A. performed experiments. G.G., J.H., and Y.L. performed computational analyses. D.H., L.C.B., S.J.E., I.G., A.S., A.A.S., R.T.F., Y.L., and D.B. supervised the experiments and computational analyses. All authors aided in the interpretation of results; read, reviewed, and approved the manuscript for publication.”

Competing interests now reads:

“G.R., Y.Y., B.A., B.M., J.W., G.G., J.H., A.A.A., A.A.S., Y.L., and D.B. are current employees of, and hold equity interest in, CAMP4 Therapeutics Corporation. D.H. and S.J.E. are current employees of Biogen Inc. L.C.B. is a stockholder and former employee of Biogen Inc. I.G., A.S., and R.T.F. are former employees of CAMP4 Therapeutics Corporation.”

The original Article has been corrected.

